# Arbutin and decrease of potentially toxic substances generated in human blood neutrophils

**DOI:** 10.2478/intox-2014-0028

**Published:** 2015-03-04

**Authors:** Jana Pečivová, Radomír Nosál', Klára Sviteková, Tatiana Mačičková

**Affiliations:** 1 Institute of Experimental Pharmacology & Toxicology, Slovak Academy of Sciences, SK-84104 Bratislava, Slovakia; 2 National Transfusion Service, SK-83101 Bratislava, Slovakia

**Keywords:** arbutin, carvedilol, reactive oxygen species, degranulation, neutrophil-blood platelet interactions

## Abstract

Neutrophils, highly motile phagocytic cells, constitute the first line of host defense and simultaneously they are considered to be central cells of chronic inflammation. In combination with standard therapeutic procedures, natural substances are gaining interest as an option for enhancing the effectiveness of treatment of inflammatory diseases. We investigated the effect of arbutin and carvedilol and of their combination on 4β-phorbol-12β-myristate-13α-acetate- stimulated functions of human isolated neutrophils. Cells were preincubated with the drugs tested and subsequently stimulated. Superoxide (with or without blood platelets, in the rate close to physiological conditions [1:50]) and HOCl generation, elastase and myeloperoxidase release were determined spectrophotometrically and phospholipase D activation spectrofluorometrically. The combined effect of arbutin and carvedilol was found to be more effective than the effect of each compound alone.

Our study provided evidence supporting the potential beneficial effect of arbutin alone or in combination with carvedilol in diminishing tissue damage by decreasing phospholipase D, myeloperoxidase and elastase activity and by attenuating the generation of superoxide and the subsequently derived reactive oxygen species. The presented data indicate the ability of arbutin to suppress the onset and progression of inflammation.

## Introduction

Neutrophils are an important part of the innate host defense. Granules from viable human neutrophils contain a variety of enzymes known to be released into the extracellular milieu as a result of stimulation by natural (Wachtfogel *et al.*, 1983) as well as artificial stimuli (Pečivová *et al.*, 2004a). Elastase may be responsible for proteolysis of vital structures (Greene & McElvaney, 2009). Its release during blood coagulation independently of thrombin suggests participation of kallikrein in neutrophil activation and expands the appreciation of the role of elastase in human diseases (Wachtfogel *et al.*, 1983). In the physiology of living systems, reactive oxygen species (ROS) and myeloperoxidase (MPO) are known to play a role which can be both beneficial (microbicidal; cellular signalling systems) and deleterious (damage of surrounding tissues; ability to delay intrinsic apoptosis and thereby the resolution of inflammation). In chronic inflammatory settings (as atherothrombotic disease, rheumatoid arthritis), neutrophils are able to interact with the endothelium, platelets and cells of the immune system, resulting in regulation of their functions (Cascão *et al.*, 2010). Recent findings have extended the role of platelets, showing their involvement in the interface between innate and acquired host defense (Von Hundelshausen & Weber, 2007). Mutual interactions of blood platelets and neutrophils are crucial in normal physiological functions as well as in the cascade of biochemical events propagating and maturating the inflammatory response resulting in serious diseases.

Carvedilol (CAR) is a unique cardiovascular drug in clinical use for treatment of hypertension, heart failure and coronary artery diseases. It possesses selective α1- and non selective β1- and β2-adrenoreceptor-blocking activities, antioxidant properties, and a potential for myocardial and vascular protection (Dulin & Abraham, 2004) as well as intense antiaggregatory activity *in vitro* (Petríková *et al.*, 2002). It was found to inhibit luminol-enhanced chemiluminescence of whole human blood *in vitro* (Nosál' *et al.*, 2005) and to decrease superoxide generation and MPO release from isolated human neutrophils activated with FMLP (Pečivová *et al.*, 2007).

In combination with standard therapeutic procedures, natural substances are gaining interest as an option to enhance the effectiveness of treatment of inflammatory degenerative diseases. Plant-based extracts are usually well tolerated, have less severe side effects and synergistic or additive pharmacological effects (Rios *et al.*, 2005; Matsuda *et al.*, 1991). The compound of bearberry leaf (*Arctostaphyllos uva-ursi*), arbutin (ARB), a hydroquinone derivative, is able to protect plants against free radical mediated and enzymatic membrane lysis (Oliver *et al.*, 1996). Besides its antiseptic and diuretic properties, ARB was reported to possess anti-inflammatory (Lee & Kim, 2012) and antioxidant effects (Takebayashi *et al.*, 2010) and the ability to decrease leukocyte infiltration and MPO activity in the mouse skin model of tumour promotion (Nakamura *et al*., 2000). ARB was found to potentiate the antiinflammatory effects of indomethacin and corticosteroids (Rios *et al.*, 2005; Matsuda *et al.*, 1991), to decrease radical formation in human neutrophils *in vitro* and in experimental arthritis (Jančinová *et al.*, 2007) and to decrease enzyme release from human neutrophils *in vitro* (Pečivová *et al.*, 2011; Pečivová *et al.*, 2012). ARB was reported to have antioxidant and other beneficial effects on the rat model of heart and mesenteric ischaemia-reperfusion (Brosková *et al.*, 2012).

In this study the impact of carvedilol, arbutin and their combination on ROS generation and degranulation was studied on the model of PMA-stimulated isolated neutrophils.

## Material and methods

Arbutin (hydroquinone β-D-glucopyranoside), Dextran T500, PMA (4β-phorbol-12β-myristate-13α-acetate), taurine, o-dianisidine were from Sigma–Aldrich Chemie GmbH (Steinheim, Germany), Lymphoprep (density 1.077 g/ml) from Nycomed Pharma (Oslo, Norway), Carvedilol from Zentiva (Prague, Czech Republic), Amplex^®^ Red Phospholipase D Assay Kit from Invitrogen (Eugene, Oregon, USA). All other chemicals used were of analytical grade. This work was approved by the Local Ethic Committee, Institute of Experimental Pharmacology and Toxicology SASc No.03/2011.

### Isolation of blood platelets

Isolation of blood platelets was performed according to Nosál' *et al.* (2000). Briefly, fresh blood was obtained at the blood bank by venepuncture from healthy male donors (20–50 years) without any medication for at least 7 days. Blood was anticoagulated with 3.8% trisodium citrate in the ratio 9:1 and centrifuged at 260 × *g* for 15 min. Platelet rich plasma (PRP) was removed, mixed with a solution containing 4.5% citric acid and 6.6% glucose (50 µl/ml PRP), and centrifuged at 1 070 × *g* for 10 min. Platelets were resuspended in an equal volume of Tyrode's solution (136.9 mmol/l NaCl, 2.7 mmol/l KCl, 11.9 mmol/l NaHCO_3_, 0.4 mmol/l NaH_2_PO_4_.2H_2_O, 1 mmol/l MgCl_2_.6H_2_O, and 5.6 mmol/l glucose) containing 5.4 mmol/l EDTA, pH 6.5. After 10-min stabilisation, the suspension was centrifuged for 6 min at 1 070 × *g* and platelets were resuspended in the same buffer without EDTA (pH 7.4) to obtain 5 × 10^5^ platelets/µl.

### Isolation of neutrophils

After removal of PRP, the blood volume was reconstituted with 0.9% NaCl. Erythrocytes were allowed to sediment in 3% dextran solution (blood:dextran 2:1) at 1 × *g* for 25 min, at 22 °C. The leukocyte/dextran mixture was centrifuged at 500 × *g* for 10 min and the pellet was resuspended in phosphate-buffered saline (PBS:137 mmol/l NaCl, 2.7 mmol/l KCl, 8.1 mmol/l Na_2_HPO_4_, and 1.5 mmol/l KH_2_PO_4_, pH 7.4). Leukocyte suspension was layered on Lymphoprep and centrifuged 30 min at 500 × *g*. Contaminating red blood cells were removed by hypotonic lysis. After centrifugation at 500 × *g*, 10 min, neutrophils were washed in PBS and resuspended in calcium- and magnesium-free PBS buffer in the final concentration of 1 × 10^7^/ml cells.

### Superoxide determination

Superoxide formation was measured in isolated human neutrophils or in the mixture of neutrophils and blood platelets as superoxide dismutase inhibitable reduction of cytochrome c. Suspension (1 × 10^6^ neutrophils or mixture neutrophils: blood platelets [1:50] / PBS with 0.9 mmol/l CaCl_2_, 0.5 mmol/l MgCl_2_) was preincubated for 5 min at 37°C with CAR and/or ARB [0.1–100 µmol/l] and subsequently stimulated by PMA [1 µmol/l] for 15 min at 37 °C. Controls were included for the effect of the stimulus, of CAR and ARB on cytochrome c reduction. After centrifugation 4 200 × *g* for 4 min at 4 °C, absorbance was measured at 550 nm in a microplate spectrophotometer (Labsystem Multiscan RC, MTX Labsystems, Inc., Vienna, Wyoming, USA).

### HOCl determination

The oxidant formed by stimulated neutrophils (10^6^ cells/sample) was measured as taurine chloramine, capable of oxidising 5-thio-2-nitrobenzoic acid (Labsystem Multiscan RC, MTX Labsystems, Inc., Vienna, Wyoming, USA) at 412 nm, according to (Weiss *et al.*, 1982).

### Myeloperoxidase release

For determination of MPO release, neutrophils were preincubated with cytochalasin B [5 µg/ml] for 5 min at room temperature. Then the neutrophils (2x10^6^/sample) were preincubated with CAR and/or ARB [0.1–100 µmol/l] in a shaker bath at 37 °C for 5 min, followed by 15-min exposure to PMA. The activity of MPO was assayed in the supernatant after centrifugation 983 × *g* for 10 min at 4 °C by determining the oxidation of o-dianisidine in the presence of hydrogen peroxide in a microplate spectrophotometer (Labsystem Multiscan RC, MTX Labsystems, Inc., Vienna, Wyoming, USA) at 450 nm.

### Elastase release

The elastase activity was assessed in the neutrophil supernatant (10^6^ cells/sample) by release of *p*-nitroaniline from MeO-Suc-Ala-Ala-Pro-Val-*p*-nitroanilide used as elastase substrate (Nakajima *et al.*, 1979). The concentration of *p*-nitroaniline was measured (Labsystem Multiscan RC, MTX Labsystems, Inc., Vienna, Wyoming, USA) at 405 nm.

### Phospholipase D activity

We measured phospholipase D (PLD) activity by using the Amplex^®^ Red PLD Assay Kit. It provides a sensitive method for measuring PLD activity *in vitro*. In this enzyme-coupled assay, PLD activity is monitored indirectly using 10-acetyl-3,7-dihydrophenoxazine (Amplex Red reagent), a sensitive fluorogenic probe for H_2_O_2_. Fluorescence was measured on a microplate spectrofluorometer (TECAN infinite 200, Tecan Group Ltd. Männedorf, Switzerland) by excitation at 530 nm and emission detection at 590 nm.

### Statistical analysis

Statistical significance of differences between means was established by Student's t-test and *p-*values below 0.05 were considered statistically significant.

## Results

Isolated human neutrophils were stimulated by a soluble stimulus, PMA, which bypasses membrane receptors and activates NADPH-oxidase via protein kinase C (PKC).

Neither ARB nor CAR [0.1–100 µmol/l] had a significant effect on superoxide generation and enzyme release from unstimulated human isolated neutrophils. To compare the effects of the drugs tested, we expressed the results as p.c. of stimulus (control = 100%).

CAR or ARB alone dose dependently [0.1–100 µmol/l] decreased superoxide generation in neutrophils (results not shown).

**Figure 1:**
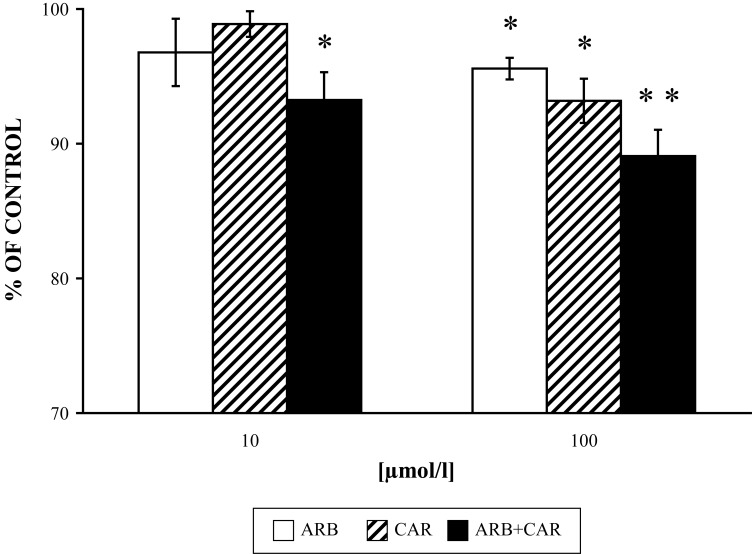
Effect of ARB and CAR and their combination (ARB+CAR) on PMA [1µmol/l 15 min/37 °C] stimulated superoxide generation versus control without the drug. Results are mean ± SEM, n=6, *p≤0.05, **p≤0.01. Absolute average control value of superoxide generation was for PMA: 50.41±1.78 nmoles per 10^6^ neutrophils/min.

Figure 1 shows the lowered effect of each drug tested alone and the combined, more pronounced decreasing effect of ARB and CAR on PMA-stimulated superoxide generation in the mixture of neutrophils:platelets (1:50).

**Figure 2:**
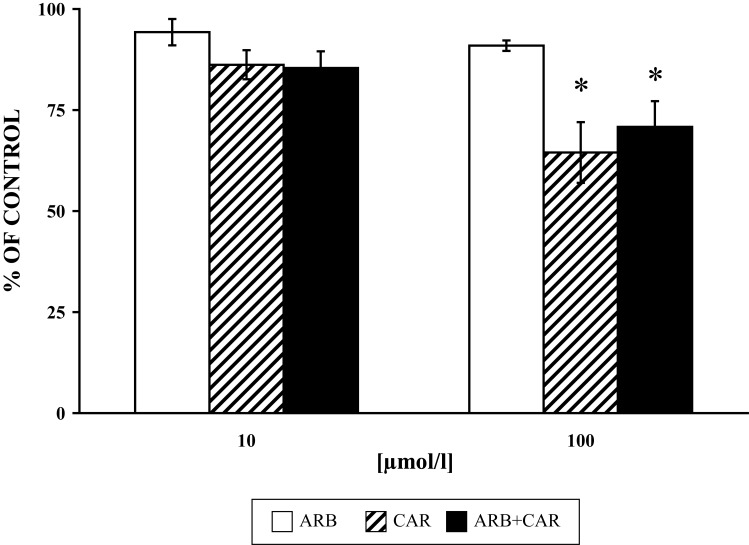
Effect of ARB and CAR and their combination (ARB+CAR) on PMA [1µmol/l 15 min/37 °C] stimulated myeloperoxidase release from neutrophils versus control without the drug. Results are mean ± SEM, n = 6, *p≤ 0.05. Absolute average control value for PMA: 3.09±0.295 ΔA/Δt (calculated as area under the curve).

**Figure 3:**
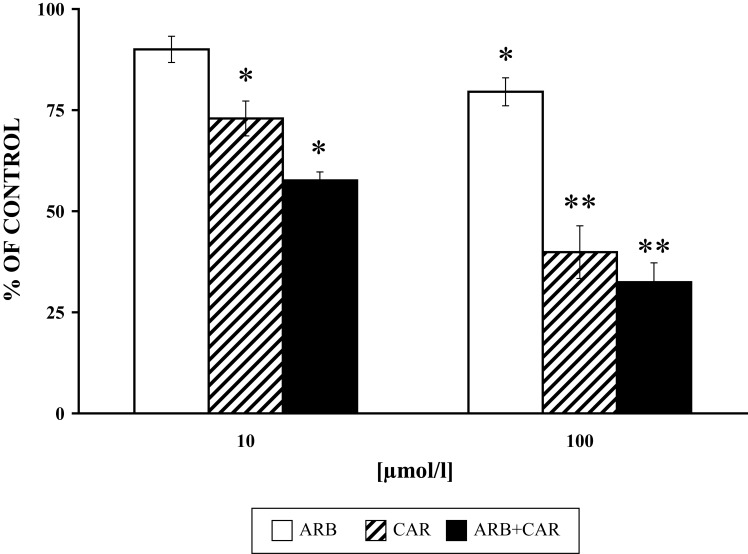
Effect of ARB and CAR and their combination (ARB+CAR) on PMA [1µmol/l 15 min/37 °C] stimulated elastase release from neutrophils versus control without the drug. Results are mean ± SEM, n=6, *p≤0.05, **p≤0.01. Absolute average control value for PMA: 18.67±0.98 mU/10^6^ cells.

**Figure 4:**
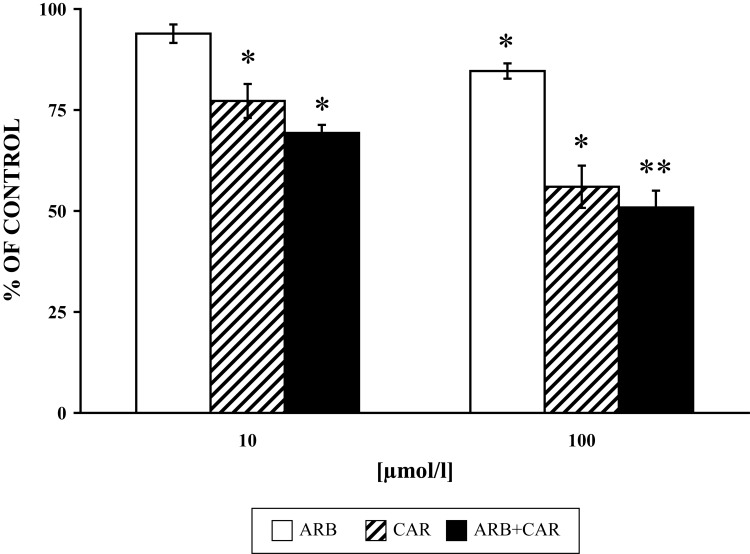
Effect of ARB and CAR and their combination (ARB+CAR) on PMA [1µmol/l 15 min/37 °C] stimulated PLD activity in neutrophils versus control without the drug. Results are mean ± SEM, n=6, *p≤0.05, **p≤0.01. Absolute average control value for PMA: 7178.5±510.83 (Relative fluorescence).

The respective effects of CAR alone, ARB alone and their cumulative effect on MPO, elastase and PLD are seen in Figure 2–4. The effects of the lower concentrations of the drugs tested are not shown since there was no significant effect on the parameters studied. ARB, CAR in the 100 µmol/l concentration and their equimolar combination decreased MPO to 90.93±3.47%, 64.5±7.5% and 73.6±6.4%, respectively. MPO-derived HOCl was inhibited by ARB, CAR in the 100 µmol/l concentration and their equimolar combination by 15.89±4.1%, 36.24±2.6% and 42.34±4.3%, respectively.

## Discussion

Neutrophils, highly motile phagocytic cells, constitute the first line of host defense, and simultaneously are considered to be central cells of chronic inflammation.

Acute inflammation is a protective highly coordinated sequence of events involving a large number of molecular, cellular and physiological changes. In a typical acute inflammatory response, effective clearance of microbial infections and/or damaged tissue is followed by resolution. This involves the elimination of neutrophils via programmed cell death, their phagocytosis by monocyte-derived macrophages that have been recruited to the inflamed site following neutrophil influx, and in turn the clearance of these macrophages. If defects arise during any part of this highly conserved pathway, inflammation will persist and become chronic (Stables & Gilroy, 2011).

Chronic inflammatory diseases are characterised by overrecruitment of leukocytes, their interactions with endothelial and other inflammatory cells resulting in increased ROS generation, enzyme release and secretion of inflammatory cytokines and chemokines (Cascão *et al.*, 2010; Serhan *et al.*, 2008; Von Hundelshausen and Weber, 2007). Persistent and excessive activation of neutrophils, along with delayed apoptosis, can intensify the risk of tissue damage and have the potential to maintain permanent inflammation. Downregulation of activated neutrophil functions may thus significantly potentiate the effectiveness of standard therapy of diseases associated with chronic inflammation and reduce undesired effects.

In combination with standard therapeutic procedures, natural substances are gaining interest as an option to enhance the effectiveness of treatment of inflammatory diseases.

In this study, we measured superoxide generation, elastase and MPO release, HOCl generation, and PLD activity, factors able to participate during pathophysiological conditions in the disturbance of successful apoptosis and resolution of inflammation.

To mimic inflammatory conditions, we tested the potential protective effect of ARB and CAR alone and in combination on the model of PMA-activated human neutrophils. In our experimental conditions, CAR dose-dependently decreased superoxide generation, similarly as recorded for fMLP-, PMA- and OZ- stimulated neutrophils (Asbrink *et al.*, 2000; Yue *et al.*, 1992; Mačičková *et al.*, 2007). In human neutrophils, CAR interfered *in vitro* and *ex vivo* both with ROS generation and with already generated ROS, suggestive of its preventive and therapeutic effect (Drábiková *et al.*, 2006), yet it was shown to be a poor scavenger of superoxide, as proven in a cell-free system (Asbrink *et al.*, 2000; Nosál' *et al.*, 2005).

ARB dose-dependently decreased superoxide generation of isolated neutrophils in our experiments (results not shown), as well as chemiluminescence in whole human blood and external oxidant generation without affecting oxidative burst arising inside isolated human neutrophils (Jančinová *et al.*, 2007).

Platelets play an important pro-inflammatory role, associated with adhesion of platelets to leukocytes and the vessel wall. The interaction of platelets with neutrophils promotes the recruitment of neutrophils into inflammatory tissue (Von Hundelshausen & Weber, 2007).

The effect of ARB and CAR on the decrease of PMA-stimulated superoxide generation was lower in the mixture of neutrophils and blood platelets than in isolated neutrophils alone, and significant only in the higher concentration [100 µmol/l] used, yet the combination of the two drugs tested resulted in inhibition also in the lower concentration of CAR [10 µmol/l] (Figure 1). We ascribed the lower inhibitory effect of ARB and CAR on superoxide generation compared to their effects on chemiluminescence (Nosál' *et al.*, 2005) to their ability to “scavenge” free radicals derived from superoxide, since this ability of the drugs tested was reported also for other experimental models (Takebayashi *et al.*, 2010; Lee & Kim, 2012) and was indicated by inhibition of MPO-derived HOCl in our experiments.

*In vivo*, blood platelets, accumulated and activated simultaneously with neutrophils, liberate substances that can eliminate or decrease the generation of oxygen metabolites, i.e. serotonin, β-thromboglobulin, adenosine and/or AMP. Mutual platelet-neutrophil interactions can weaken the effect of drugs (Ward *et al.*, 1988; Pečivová *et al.*, 2004b; Číž *et al.*, 2007).

The inhibitory effect of CAR on superoxide generation and enzyme release from PMA-stimulated neutrophils in higher concentrations (10 and 100 µmol/l), in contrast to lower concentrations (Pekarová *et al.*, 2009), indicates the possibility that CAR, similarly to other lipophilic β-adrenoceptor/blocking drugs, interferes with membrane structure, influencing predominantly phospholipid metabolism. Physicochemical properties of CAR (partition coefficient, dipole moment, and high lipophilicity) and its antiplatelet activity (Petríková *et al.*, 2002) support this conclusion.

Phospholipases C and D have been linked to the antimicrobial functions of reactive oxidant generation, granule secretion, and phagocytosis (Gomez-Cambronero & Keire, 1998). Exposure of many cell types to PMA stimulates PLD activity, thereby implying an action by PKC. PLD hydrolyses phosphatidylcholine to phosphatidic acid (PA) and choline, where PA is considered to be the main effector of PLD function in cells. PA is reported to function as a second messenger, involved in membrane protein recruitment and membrane fusion processes, and PLD is proposed to play a role in signalling, intracellular transport, cytoskeletal rearrangements and apoptosis (Nozawa, 2002).

Since in our experiments ARB inhibited PMA-stimulated but not the spontaneous superoxide generation and chemiluminescence (Jančinová *et al.*, 2007), it seems to interact with some processes involved in activation of neutrophil oxidative burst.

We studied the effect of ARB, CAR and their combination on PLD activity, MPO and elastase release from granules of PMA-stimulated neutrophils. A higher inhibitory effect of CAR on MPO release from intact cells was recorded in comparison to its direct effect on enzyme activity (Pečivová *et al.*, 2007). Both drugs decreased MPO and elastase release, and applied together, their effect was more pronounced (Figure 2 and 3). The same “trend” was measured for PLD activity (Fig. 4). Based on the structural similarity of ARB to a known inhibitor of phospholipase A_2_, it appeard conceivable that inhibition of phospholipases (Oliver *et al.*, 1996) or interference with phospholipid derived mediators (Stables & Gilroy, 2011) may be possible mechanisms involved.

Neutrophil apoptosis is one of the critical determinants of the outcome of the inflammatory response (Filep & El Kebir, 2009). Rapid induction of apoptosis in activated neutrophils and their subsequent removal from inflammatory sites by macrophages may prevent further damage to healthy tissues caused by ROS generation and release of toxic granule contents.

MPO triggers elastase and MPO from the granules and upregulates surface expression of Mac-1 on neutrophils, implying an autocrine and paracrine mechanism for perpetuation of the inflammatory response (Filep & El Kebir, 2009; Lau & Baldus, 2005). At sites of inflammation, MPO may function as a catalytic sink for NO, influencing its bioavailability (Abu-Soud & Hazen, 2000), and moreover, it is implicated to prolong the life span of neutrophils, thereby delaying the resolution of inflammation (Filep & El Kebir, 2009; Lau & Baldus, 2005).

Apoptotic neutrophils display changed morphological and biochemical characteristics and show molecular alterations on their cell surface. Resolution of inflammation is normally associated with orderly removal of dying apoptotic inflammatory cells by phagocytes through cell recognition receptors. Phosphatidylserine is a molecular marker whose externalisation facilitates the recognition of apoptotic neutrophils by macrophages (Akgul *et al.*, 2001).

Flow cytometry demonstrated that neutrophil elastase cleaved the phosphatidylserine receptor, implying a potential mechanism for delayed apoptotic cell clearance *in vivo* (Vandivier *et al.*, 2002).

The neutrophil serine protease elastase occupies an important position at the apex of a specific protease hierarchy. It has a number of important intrinsic proteolytic properties, it behaves also as a proinflammatory mediator, and in certain circumstances controls signalling mechanisms regulating innate immunity (Greene & McElvaney, 2009).

ARB was reported to have the potency to weaken priming of neutrophils during the inflammatory process, to decrease the concentration of radicals generated extracellularly by neutrophils, and that without affecting the formation of intracellular radicals essential for regulatory and microbicidal functions (Jančinová *et al.*, 2007). Moreover, ARB has been suggested to regulate adhesion and trafficking of leukocytes and to attenuate LPS induced expression of inflammatory genes, such as iNOS, IL-1β, and TNF-α by downregulation of NF-κB (Lee & Kim, 2012).

The pluripotency of MPO and elastase distinguishes them as unique factors controlling many aspects of infection and inflammation. They are implicated as participants involved in direct cytotoxicity as well as in the perpetuation of inflammation. Thus the ability of ARB to decrease their free availability, along with reducing ROS generation, may significantly potentiate the effectiveness of therapy of diseases associated with chronic inflammation, reducing at the same time undesired effects of the antiinflammatory drugs used.

The presented data, together with those reported in the literature, indicate the beneficial ability of ARB to suppress the onset and progression of inflammation.
